# Adaptive signal coloration maintained in the face of gene flow in a Hispaniolan *Anolis* Lizard

**DOI:** 10.1186/s12862-016-0763-4

**Published:** 2016-09-20

**Authors:** Julienne Ng, Alison G. Ossip-Klein, Richard E. Glor

**Affiliations:** 1 Department of Ecology and Evolutionary Biology, University of Colorado, Boulder, CO 80309 USA; 2 Department of Biology, University of North Georgia, Gainesville, GA 30503 USA; 3 Biodiversity Institute and Department of Ecology and Evolutionary Biology, University of Kansas, Lawrence, KS 66045 USA

**Keywords:** Dewlap, Geographic variation, Local adaptation, Speciation, *Anolis distichus*

## Abstract

**Background:**

Studies of geographic variation can provide insight into the evolutionary processes involved in the early stages of biological diversification. In particular, multiple, replicated cases of geographic trait divergence present a powerful approach to study how patterns of introgression and adaptive divergence can vary with geographic space and time. In this study, we conduct replicated, fine-scaled molecular genetic analyses of striking geographic dewlap color variation of a Hispaniolan *Anolis* lizard, *Anolis distichus*, to investigate whether adaptive trait divergence is consistently associated with speciation, whereby genetic divergence is observed with neutral markers, or whether locally adapted traits are maintained in the face of continued gene flow.

**Results:**

We find instances where shifts in adaptive dewlap coloration across short geographic distances are associated with reproductive isolation as well as maintained in the face of gene flow, suggesting the importance of both processes in maintaining geographic dewlap variation.

**Conclusion:**

Our study suggests that adaptive dewlap color differences are maintained under strong divergent natural selection, but this divergence does not necessarily lead to anole speciation.

**Electronic supplementary material:**

The online version of this article (doi:10.1186/s12862-016-0763-4) contains supplementary material, which is available to authorized users.

## Background

Geographic trait variation is widely found within species [1]. The evolution of such variation can arise from divergent natural selection driving adaptation to local environments [2, 3], but whether adaptive trait variation persists through time depends on whether divergent selection is strong enough to counteract the homogenizing effects of gene flow [4–6]. Two alternatives may therefore result from ecologically-driven phenotypic divergence: speciation, whereby reproductive isolation has evolved, or selection may maintain a stable coexistence of two or more locally adapted phenotypes despite gene flow. Identifying the consequence of adaptive divergence is central to understanding the roles of local adaptation and speciation during the early stages of the formation of biological diversity.

Comparative studies of naturally replicated hybrid zones where adaptively divergent, closely related taxa come into contact and potentially interbreed can provide a powerful approach to study how patterns of introgression and adaptive divergence can vary with geographic space and time [7]. In particular, fine-scaled molecular genetic analyses across such replicated contact zones can directly test whether divergent natural selection necessarily leads to divergence at neutral loci (e.g. [8–11]). For example, studies of mice and lizards that adaptively vary in coloration for crypsis show that not all geographic transitions in color are associated with reduced gene flow [8, 10]. This suggests that although phenotypically different populations have yet to evolve barriers to reproduction, strong selection is acting to maintain phenotypic divergence, most likely from visual predators [12–14].

A remarkably polymorphic lizard, *Anolis distichus*, provides an ideal system to investigate whether adaptive trait divergence consistently leads to the same genetic outcome. Across Hispaniola, *A. distichus*’ dewlap (an extensible throatfan) varies adaptively in response to heterogeneous environments, whereby dewlaps tend to be larger, orange and less bright in wetter habitats, while in drier habitats, dewlaps are smaller, yellow and relatively brighter [15]. This geographic variation in dewlap color has led taxonomists to diagnose eight subspecies on mainland Hispaniola alone [16] that are characterized by deeply divergent mitochondrial DNA (mtDNA) haplotypes, and differentiation in allozyme and nuclear DNA loci [17–19]. We have also shown in previous work that in two areas of contact, subspecies with different colored dewlaps show a reduction in gene flow, consistent with the expected signature of speciation [20]. However, it remains unclear if these reduced patterns of gene flow occur at other contact zones between divergent dewlap phenotypes, or whether other phenotypic disjunctions represent cases of strong natural selection on dewlap color but with ongoing gene flow. The many other naturally replicated shifts in dewlap color across the range of *A. distichus* provide an excellent opportunity to assess how selection on an adaptive trait may be associated with patterns of introgression across space and time.

Here, we significantly expand upon prior work by conducting replicated molecular genetic analyses of nine transects that span *A. distichus* populations. We contrast results from five geographically diverse transects that extend across phenotypically distinct populations that have been diagnosed using differences in dewlap color and pattern, and four control transects that span the same dewlap color. Specifically, we aim to test whether adaptive dewlap color divergence is associated with (i) speciation, whereby genetic differentiation is observed with neutral markers. Following the general lineage concept [21, 22], this result would suggest that populations differing in dewlap color represent independently evolving lineages. Alternatively, (ii) if genetic differentiation is not observed with neutral markers, this suggests that locally adapted traits are maintained in the face of continued gene flow.

## Methods

### Sampling

**Fig. 1:**
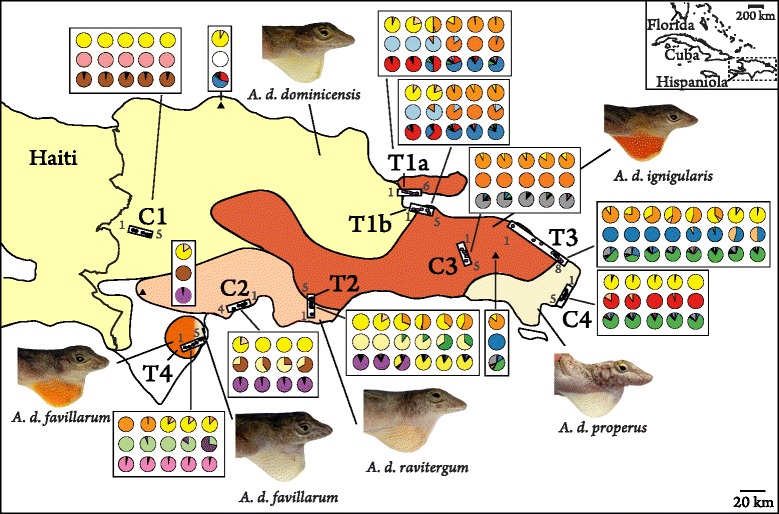
Distribution and sampled localities of *Anolis distichus* subspecies in the Dominican Republic. Colored regions demark respective range distributions and dominant dewlap color of *A. distichus* subspecies (adapted from [16]). Filled black circles surrounded by boxes represent the study transects and associated sampling localities: T1 – T4 represent transects that transition between populations with different dewlap coloration; C1 – C4 represent control transects that span populations with similar dewlap coloration. To permit comparison to other figures, the first and last sample sites of each transect are numbered. Black triangles indicate additional sampled sites outside of our transects. Pie charts represent dewlap and genetic variation for each site in numerical order from left to right (see also Fig. 2 for more details). The top row of pie charts represent dewlap color variation with yellow, orange and peach proportions indicating the average proportion of yellow, and solid or diffuse orange visually observed in the dewlap, respectively. Females or juveniles found at the site are not included as they cannot be scored for dewlap color. The middle row of pie charts represents mitochondrial haplotype variation as determined from a maximum likelihood phylogenetic analysis, with different colors representing different mitochondrial clades (see also Fig. 3). Missing haplotype data is not included. The bottom row of pie charts represents nuclear genotypic variation as determined from STRUCTURE analyses, with different colors representing different genetic clusters (*K*) (*K* = 9 was the best estimate) (see also Fig. 2 for more details). Further substructure from subsequent STRUCTURE analyses on separate clusters is not shown

We sampled nine linear transects in the Dominican Republic: five transects that spanned from populations with primarily yellow dewlaps to populations with primarily orange dewlaps (herein referred to as “transitional” transects; T1a, T1b, T2-T4, Fig. 1), and four control transects that extended across populations exhibiting the same dewlap color (C1-C4, Fig. 1). Four of the five transitional transects spanned populations previously diagnosed as different subspecies, primarily based on dewlap color differentiation [16]. The only exception was transect T4, which spanned populations assigned to a single subspecies (*A. d. favillarum*) that exhibits geographic variation in dewlap color. All four control transects involved sampling within the range of a single subspecies.

For both transitional and control transects, we aimed to sample five or six evenly spaced localities along 10–20 km linear transects (Fig. 1, Additional file 1: Table S1). For the transitional transects, this sampling scheme captured the transition in dewlap color from one dewlap color extreme to the other. Inaccessibility or unsuitable habitat determined transect orientation and also led to some variation in distance between sites (1.74 km to 8.43 km; mean ± SD = 3.26 ± 1.55 km). We previously reported results from sampling along two of these transects, T1a and T2 [20]. Here, however, we include data from an additional site between sites 2 and 3 along transect T2 that appears to be close to the point of contact between the two subspecies (T2-2.5; 0.89 and 1.55 km from sites 2 and 3, respectively). In addition, following initial sampling of T3 (T3-4 to T3-8), we subsequently extended the transect into the range of *A. d. ignigularis* with three additional sites sampled at larger intervals (T3-1 to T3-3) after preliminary analyses failed to recover evidence for geographic genetic variation. As such, these additional sites ranged from 5.28 - 19.23 km in distance from the next adjacent sites, resulting in T3 spanning a total distance of 42.20 km. All control transects spanned populations of the same subspecies and ranged in distance from 8.98 - 14.03 km, with four or five localities sampled along each transect (Fig. 1, Additional file 1: Table S1). We obtained tissue samples (either tail tips or livers) for molecular genetic analyses from a median of 20 individuals at each transect locality (sample sizes ranged from 10 to 38; Additional file 1: Table S1). In addition to transect sampling, we also sampled 20 individuals from three additional localities (herein called non-transect sites), each of which represent *A. d. dominicensis, A. d. ignigularis* and *A. d. ravitergum* elsewhere within their ranges to further investigate geographic genetic variation (Fig. 1).

### Characterizing dewlap color variation

We ascertained phenotypic variation across all transects by visually categorizing patterns of dewlap color following previous studies [18, 20]: (i) primarily yellow (<10 % orange), (ii) yellow with a small orange spot (occupying 10–40 % of the dewlap), (iii) yellow with a large orange spot (occupying 50–90 % of the dewlap), or (iv) orange with a narrow yellow margin (>90 % orange). We further categorized any orange coloration as either diffuse (blush) or solid. Assignments of each dewlap were independently assessed and verified by JN and REG. Although we focus on visual assessments of dewlap color (as yellow or orange), this has previously been shown to be consistent with spectrometer measurements of hue, which is an axis of adaptive dewlap divergence [15, 20]. In addition, while some *A. distichus* dewlaps reflect ultraviolet (UV) wavelengths, UV reflectance is not correlated with different habitat types and is therefore not considered an adaptive trait in this species [15]. Juveniles and females, which lack or have reduced dewlaps, were not scored for dewlap color.

### Characterizing genetic variation

#### Mitochondrial DNA sequencing and phylogenetic analyses

We investigated patterns of mtDNA differentiation along transects by obtaining mtDNA sequence data for all individuals sampled from sites along each transect (*N* = 955). We focused on a 1147 basepair (bp) region of mtDNA extending from the beginning of ND2 (subunit two of NADH dehydrogenase) through to tRNA^Ala^ that has been widely used in previous phylogenetic and phylogeographic studies of anoles (e.g. [23–29]). We also obtained mtDNA sequence data from this same region for individuals at the three non-transect sites as well as 195 additional individuals from 50 other *A. distichus* populations across Hispaniola, including three Hispaniolan subspecies that did not occur in any of our transects: *A. d. sejunctus*, a subspecies found on Isla Saona (an island off the south-east coast of the Dominican Republic), and two Haitian subspecies, *A. d. vinosus* and *A. d. aurifer*. To represent outgroups, we obtained previously published mtDNA haplotypes from eight species: four species from the *A. distichus* species group (*A. brevirostris*, *A. caudalis*, *A. websteri* and *A. marron*), two representatives from clades closely related to the *distichus* series (*A. cristatellus* [*cristatellus* series] and *A. bimaculatus* [*bimaculatus* series]) [30] and two more distantly related anoles (*A. punctatus* and *A. occultus*) [27]. Our complete mtDNA dataset comprised haplotypes sampled from 1305 individuals, of which 675 were unique; 332 sequences were obtained from previous studies [15, 17, 20, 27] and 973 new sequences were generated for this study (GenBank accession numbers: KX854021-KX855205).

We generated new mtDNA sequences by first extracting genomic DNA from tissue samples using the Wizard genomic DNA purification kit (Promega). Using primers L4437 [31] and H5730 [32], we conducted 25 μL PCR reactions with 1 μL genomic DNA, 2.5 μL of each 2 μM primer, 2.5 μL of New England Biolabs (NEB) 10× buffer (10 mM Tris–HCl, 50 mM KCl), 2.5 μL of 25 mM MgCl_2_, 2.5 μL of 0.5 mM dNTP solution, 0.125 μL of NEB *Taq* polymerase and 11.4 μL H_2_O. Our thermocycling protocol involved an initial denaturation at 95 °C for 4 min, followed by 30 cycles of 95 °C for 35 s, annealing at 52 °C for 35 s, and extension at 70 °C for 2 min 30 s. We sent successfully amplified PCR products to a commercial facility for purification and Sanger sequencing (Beckman Coulter Genomics, Massachusetts). We assessed each sequence chromatograph for quality in Geneious v4.6.1 [33] and used the pairwise alignment tool in MacClade 4.0 [34] to align sequences. With no indels in the protein coding sequences, alignment was straightforward. The tRNA genes were aligned using secondary structural models [35]. As 43 bp of sequence data from tRNA genes were highly variable in length and a region of apparent ambiguous alignment, we excluded these regions from our dataset prior to analysis, leaving 1104 aligned characters without gaps.

We inferred phylogenetic relationships among *A. distichus* haplotypes using a maximum likelihood (ML) approach in RAxML v7.0.3 [36] and Bayesian inference in MrBayes v3.2 [37, 38] (alignment and trees deposited in TreeBASE: study ID S19838). We used PartitionFinder v1.1.1 [39] to determine the best fitting partitioning scheme with four data blocks: a separate partition for each codon position within ND2 and a fourth partition for the tRNA region. For RAxML, PartitionFinder identified three partitions to be the best fitting scheme, with GTR + I + G to be the best fitting substitution model for the first ND2 codon position and the tRNA region merged as one partition, as well as the second ND2 codon position, while GTR + G was identified as the most appropriate model of evolution for the third ND2 codon position region. For MrBayes, which allows analyses under more evolutionary models, PartitionFinder identified HKY + I + G to be the most appropriate model of evolution for the first ND2 codon position, GTR + I + G for the second ND2 codon position and tRNA region, and GTR + G for the third ND2 codon position. For both ML and Bayesian analyses, we did not include a parameter for the proportion of invariable sites (I) since the interactions between the I and G parameters can potentially lead to inaccurate estimates due to non-independent optimization [40]. For our ML analysis, we conducted 1000 nonparametric bootstrap replicates and computed pairwise ML patristic distances from the best ML tree. For trees generated under Bayesian inference, we ran two independent analyses for 10 million generations and assessed convergence by checking the average standard deviation of split frequencies in MrBayes and using Tracer v1.6 [41]. We considered there to be genetic differentiation in mitochondrial DNA along our sampled transects if populations from either ends of the transect belonged to distinct mitochondrial clades (node support as measured by bootstrap values and posterior probabilities ≥ 75 %).

For each transect site sampled, we calculated the number of haplotypes, number of polymorphic sites, nucleotide diversity and the mean number of pairwise differences between haplotypes using ARLEQUIN v3.11. Using the same program, we also assessed genetic differentiation between populations using Φ_ST_ (an analogue of F_ST_) and tested for significant genetic differences between sites using 10,000 permutations. We also constructed a haplotype network that comprised haplotypes from all transect sites in PopART (http://popart.otago.ac.nz) using the TCS method [42].

#### Microsatellite genotyping and population structure analyses

To assess nuclear differentiation along transects, we genotyped all individuals using 7 highly polymorphic di- and trinucleotide microsatellite loci: DISTA2B12, DISTBB5, DISTBC4, DISTAG1, DISTCC7, DISTAH6 and BREV2E9 [43]. We followed the same PCR protocols as described for mtDNA sequence data (above section) but, following Ng and Glor [20], we included a fluorescently-labelled CAG primer (6-FAM, VIC or NED) at a 1:20 ratio (0.01 μM CAG-tagged forward primer and 0.19 μM fluorescent CAG primer) to enable multiplexing. We followed thermocycling conditions detailed in Ng et al. [43] and visualized products on an ABI 3730 Genetic Analyzer (Applied Biosystems) located at the University of Rochester’s Functional Genomics Center. Genotypic data were analyzed with GENEMAPPER V3.7 software (Applied Biosystems). We tested all microsatellite loci for within population departures from Hardy-Weinberg equilibrium (HWE) and linkage disequilibrium (LD) using GENEPOP v4.0 [44], and accounted for multiple tests using sequential Bonferroni correction [45].

We tested for nuclear genetic differentiation along each transect using (i) genetic distance metrics and (ii) a Bayesian clustering approach. We did not use cline analyses (e.g. [46, 47]) because our microsatellite markers are not diagnostic; they do not show fixed, or almost fixed, differences between the two subspecies along transects (see Results). We calculated pairwise estimates of Φ_ST_ between adjacent sites along each transect using ARLEQUIN v3.11 and tested for significant genetic differences between sites using 10,000 permutations. In addition, we used a genetic distance metric based on the proportion of shared alleles between populations (D_PS_) [48], calculated using MSA v4.05 [49]. D_PS_ has an advantage over Φ_ST_ in that it does not assume population equilibrium [50, 51].

We conducted Bayesian clustering analyses using the program STRUCTURE v2.3.3, which uses a Markov chain Monte Carlo algorithm to probabilistically assign individuals to different genetic clusters (*K*) [52]. We inferred genetic structure after combining data across all transects and the three non-transect sites. We used the admixture model, which allows individuals to have mixed ancestry, in addition to the correlated allele frequencies model, which accounts for potential linkage disequilibrium between markers that can arise due to admixture [53], and did not include *a priori* information about where individuals were sampled. We ran analyses for 10^5^ iterations following a burn-in period of 10^4^ iterations for each value of *K* ranging from 1–15. We chose a maximum K value of 15 because we expected that there would be at least 8 different genetic clusters across our transitional transects if genetic differentiation is associated with adaptive dewlap color transition (or at least 10 if individuals of the same subspecies were genetically different along T1a and T1b). We ran 10 independent runs of *K* to ensure consistent probability estimates and that particular runs were not trapped in different modes in the parameter space [52]. The average of all ten runs were subsequently used to assess genetic structure. To identify the best estimate of *K*, we used log probabilities Pr(X|*K*) and calculated Δ*K* [54] using the web-based program STRUCTURE HARVESTER [55]. We also investigated whether there was more subtle population structure by repeating the analysis on each genetic cluster identified. Following protocols outlined in Coulon et al. [56], we assigned individuals to a particular genetic cluster if their inferred ancestry to that group was higher than 0.6. With each genetic cluster, we sequentially ran STRUCTURE analyses using the same parameters as the initial run and continued this process until the optimal *K* was 1.

We interpreted results of distinct nuclear genotypic clusters at either ends of the transect to be evidence for neutral genetic divergence. We diagnosed how far along populations were in the speciation continuum by the frequency of admixed individuals. If geographic genetic structure was evident but the frequency of admixed individuals was high and widely spread across different populations, we considered this to reflect low levels of restricted gene flow, and that the populations were at an earlier stage in the speciation continuum. In contrast, a limited number or no evidence of admixed individuals indicated a strong reduction of gene flow, likely representing populations in the later stages of speciation.

## Results

### Dewlap color variation

**Fig. 2:**
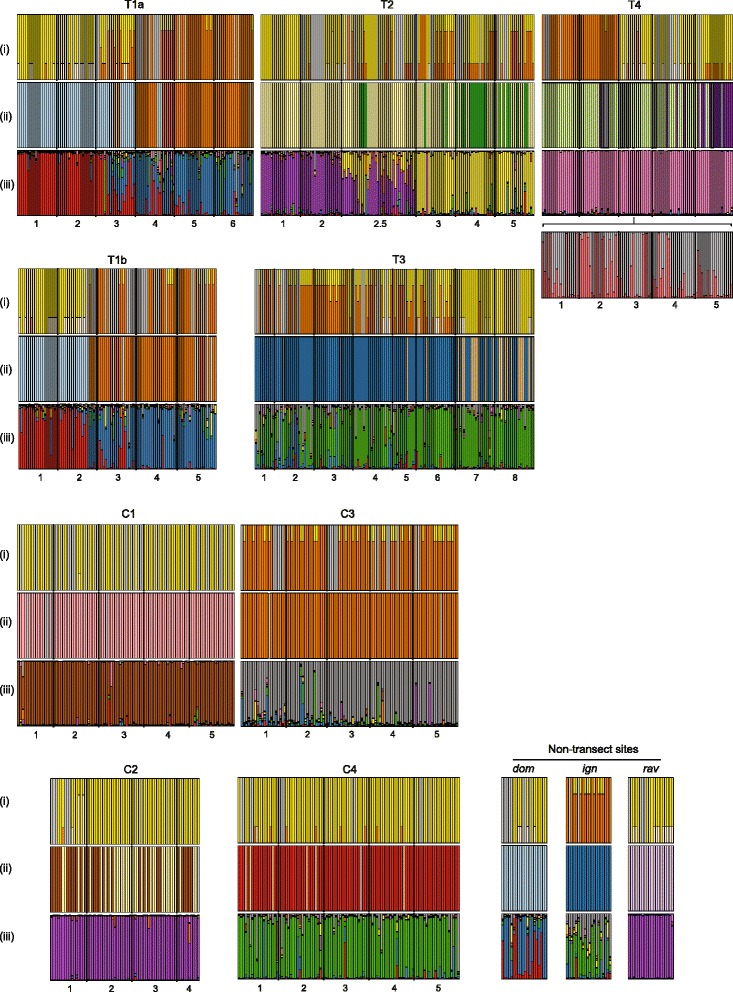
Phenotypic and genetic variation across transitional transects (T1-4), control transects (C1-4) and non-transect sites. Vertical bars in each graph represent individuals and vertical black lines demark different sample sites. Sample site numbers are shown underneath each set of graphs. **i** Dewlap color variation with colors representing percentages of orange and yellow in the dewlap. Peach colored bars represent a diffuse orange while grey bars represent females or juveniles, which lack or have reduced dewlaps and thus were not scored for color. **ii** Mitochondrial haplotype variation as determined from a maximum likelihood phylogenetic analysis. Different colored bars represent haplotypes belonging to different clades (see also Fig. 3). Grey bars represent missing haplotype information. **iii** Nuclear genotypic variation as determined from STRUCTURE analyses (*K* = 9). Each color represents a distinct genetic cluster, with different colors within each bar representing the proportion of the individual’s genotype assigned to the cluster. Proportions shown are averages from 10 replicate runs. The additional graph shown under T4 indicates substructure revealed with additional STRUCTURE analyses following [56]

Along all transitional transects, we confirmed the presence of a phenotypic transition in dewlap color and pattern. At one side of each transect, males exhibited dewlaps that were completely yellow or had a small proportion of orange, while towards the other side of the transect, we found males with a higher proportion of orange in their dewlaps (Figs. 1 and 2). The nature of the shift between yellow and orange dewlapped populations, however, varied among transitional transects. For example, T1b and T4 exhibited an abrupt shift in the proportion of orange found in the dewlap; populations characterized by largely yellow or largely orange dewlaps were separated by only approximately 3 km and there was little evidence for intermediate amounts of orange at any of the sampled localities (Figs. 1 and 2). Meanwhile, T1a and T3 exhibited a more gradual cline in the amount of orange in the dewlaps; populations having almost entirely orange or yellow dewlaps were separated by 6.65 and 20.58 km respectively, and one or more localities included individuals with intermediate amounts of orange in their dewlaps (Figs. 1 and 2). Along the remaining transitional transect, T2, dewlaps were rarely entirely or almost entirely orange, but the presence of some orange in the dewlap transitioned from being uncommon to common over a relatively short distance (0.89 km) (Figs. 1 and 2).

We found similar dewlap phenotypes across each control transect. All males sampled along C3 had dewlaps with greater than 60 % orange, while individuals sampled along all other control transects had very little orange in the dewlaps; the two individuals at C2-1 that exhibited greater than 50 % orange in the dewlap was diffuse in coloration (Figs. 1 and 2).

### Mitochondrial phylogenetic relationships

**Fig. 3:**
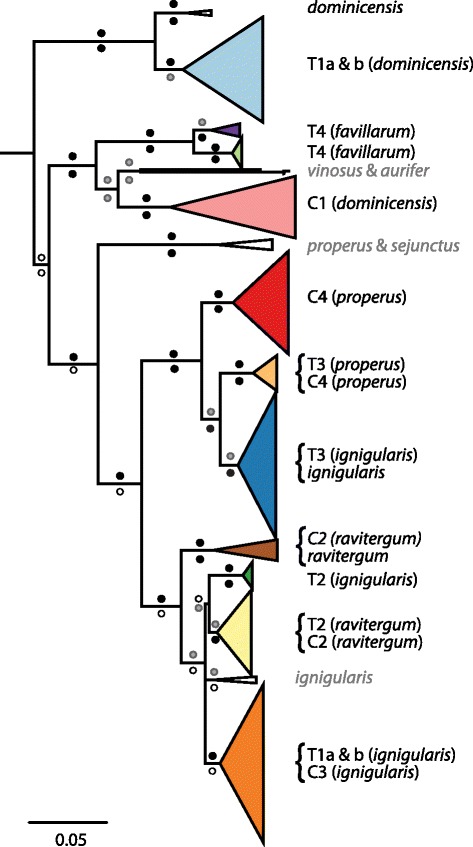
Maximum likelihood phylogeny inferred by RAxML from *Anolis distichus* mtDNA haplotypes. Haplotypes used as outgroups have been pruned from this tree. Colored triangles represent clades and are labelled with the transect and subspecies from which the haplotype was sampled (note that for transitional transects, the expected subspecies collected is listed based on locality along the transect). Labels in black font without transect IDs represent non-transect sampled sites. Clades labelled in grey font represent haplotypes that were sampled from additional sites that were included in the phylogenetic analysis but not used in other aspects of this study. Circles above and below branches represent node support measured as bootstrap values and posterior probabilities from RAxML and MrBayes, respectively: black ≥90 %, grey ≥75 %, white <75 %

Phylogenetic reconstructions under ML and Bayesian inference were largely congruent with node support greater or equal to 75 % for major groups (Fig. 3), with the exception that the ML tree showed strong support for a clade comprising *A. d. ignigularis* haplotypes from T1a, T1b and C3, while the Bayesian consensus tree showed strong support for a separate clade for each transect. As this incongruence does not affect our overall conclusions, we here only discuss and report clade membership for *A. d. ignigularis* based on to the ML tree for brevity.

**Fig. 4:**
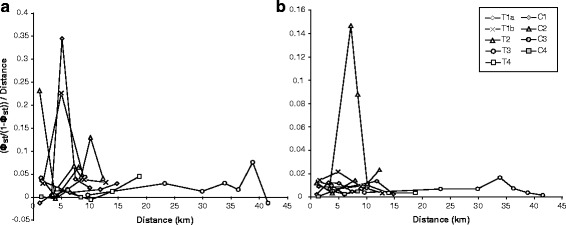
Pairwise estimates of Φ_ST_ in (**a**) mtDNA and (**b**) microsatellite loci between sites sampled along transitional transects (T1-T4; unfilled shapes) and control transects (C1-C4; filled shapes). Φ_ST_ estimates were adjusted for distance between sites using linearized genetic distance (Φ_ST_/(1-Φ_ST_)) divided by geographic distance (km)

Along each transitional transect, we found haplotypes from two different mitochondrial clades (Figs. 1, 2 and 3). Along T1a and T1b, most of the haplotypes sampled from either end of the transects showed deep divergence; haplotypes along both T1 transects belonged to deeply divergent clades (15.1 % and 15.8 % average uncorrected sequence divergence, respectively) and were also divergent in the haplotype network (Additional file 1: Figure S1). Haplotypes from the western side of the transects grouped with other *A. d. dominicensis* haplotypes while haplotypes from the eastern side grouped with other *A. d. ignigularis* haplotypes (Fig. 3). This differentiation was also reflected in significant Φ_ST_ estimates, particularly between sites T1a-3 and 4 (Φ_ST_ = 0.75; *P* < < 0.001) and sites T1b-2 and 3 (Φ_ST_ = 0.60; *P* < < 0.001) which were each at least 4 times greater than any other adjacent sites along the same transect, after correcting for differences in geographic distance (Fig. 4). A few haplotypes sampled from the middle and more eastern sites were assigned to the same clade of *A. d. dominicensis* haplotypes as the haplotypes sampled from the westernmost sites, suggesting limited mtDNA introgression (Figs. 1 and 2). The middle sites of T1a and b also had greater mtDNA genetic diversity (Additional file 1: Table S2). All other transitional transects (T2-T4) exhibited relatively less genetic differentiation in mtDNA (T2-T4: 3.7 %, 4.0 % and 5.0 % average uncorrected sequence divergence, respectively) as well as evidence of mtDNA introgression (Figs. 1 and 2). Mitochondrial genetic diversity estimates reflected these patterns whereby we found greater mtDNA haplotype and nucleotide diversity at sites that showed mtDNA introgression (Additional file 1: Table S2).

Along the control transects, C1 and C3 comprised mtDNA haplotypes from the same clade (Figs. 1, 2 and 3). We found haplotypes from two closely related clades along C2 and C4 (4.7 % and 5.3 % average uncorrected sequence divergence, respectively) but these haplotypes did not show any geographic structure (Figs. 1 and 2).

Assessing overall phylogenetic relationships among *A. distichus* mtDNA haplotypes, we found *A. d. favillarum* to be the only monophyletic Dominican subspecies from mainland Hispaniola. Mitochondrial haplotypes from the other four Dominican *A. distichus* subspecies from mainland Hispaniola (*A. d. dominicensis*, *A. d. ignigularis*, *A. d. properus* and *A. d. ravitergum*) did not form monophyletic groups (Fig. 3). Instead, *A. d. ravitergum* and *A. d. properus* sampled along our transects were each rendered paraphyletic by *A. d. ignigularis* sampled along the same transects (T2 and T3 respectively). Our finding that most *A. distichus* subspecies are non-monophyletic is inconsistent with other phylogenetic studies of the group [17, 19], but is likely due to our inclusion of individuals from subspecific contact zones where mitochondrial introgression can confound phylogenetic placement. Our phylogeny, however, is consistent with Geneva et al. [19] in showing well-supported haplotype clades within *A. d. dominicensis* that appear to be geographically structured; haplotypes from transect C1 in central Hispaniola are most closely related to haplotypes sampled from *A. d. favillarum* and Haitian *A. distichus* subspecies, while *A. d. dominicensis* haplotypes sampled from northern Dominican Republic (T1a and b, non-transect *A. d. dominicensis* site) formed a separate group (Fig. 3).

### Microsatellite loci tests of HWE and LD

The microsatellite loci exhibited significant deviations from HWE in 57 of 364 intrapopulation assessments (Additional file 1: Table S1). All loci but DISTAH6 deviated from HWE in only a few populations (3–10 populations), while DISTAH6 deviated from HWE in 23 of 57 sample sites. Thus, we ran analyses using all seven loci and then repeated analyses without genotypic data from DISTAH6. As analyses did not qualitatively differ, we here report results of analyses on all seven loci. We detected little evidence of LD among microsatellite loci; in 6 of 52 sampled sites, one pair of loci showed significant LD after Bonferroni correction (*P* < 0.007). These pairs differed between sites, except for DISTCC7 and DISTAH6, which showed significant LD in two sites along T4 (T4-1 and T4-3).

### Patterns of nuclear genetic structure

The results of our STRUCTURE analysis identified *K* = 9 to be the best estimate of *K*. Of the transitional transects, three showed that individuals on either side of the transect belonged to different genetic clusters (T1a and b, T2; Figs. 1 and 2). Sampled sites in the middle of these transects included individuals from both genetic clusters as well as admixed individuals. While at least three localities included admixed individuals along T1a and T1b, we only found admixed individuals at one site along T2 (T2-2.5). The other two transitional transects (T3 and T4) did not show any genetic structure despite T3 being the longest transect; most individuals sampled along the transect were predominantly assigned to the same cluster. Additional runs on each genetic cluster revealed two further genetic clusters along T4 but there was no obvious geographic structuring (Fig. 2). Individuals along control transects were primarily assigned to the same genetic cluster as others sampled from the same transect (Fig. 2).

While STRUCTURE analyses showed T1a, T1b and T2 to all exhibit genetic differentiation, pairwise Φ_ST_ and D_PS_ estimates showed that genetic differentiation along T1a and T1b were comparable to that found along the control transects and the transitional transects, T3 and T4, which did not exhibit genetic structure in the STRUCTURE analyses (Fig. 4, Additional file 1: Figure S2). Meanwhile, pairwise Φ_ST_ and D_PS_ estimates along T2 showed moderately high genetic differentiation between T2-2 and T2-2.5 (Φ_ST_ = 0.12; D_PS_ = 0.48) that, after correcting for differences in geographic distance, were at least 7 and 3 times greater respectively than that found along any other transect (geographic distance corrected: Φ_ST_ = 0.15; D_PS_ =0.54; Fig. 4, Additional file 1: Figure S2).

Assessing general genetic structure within subspecies, we found that subspecies that have a widely distributed range exhibited nuclear genetic structure. The most widespread of the subspecies, *A. d. dominicensis*, comprised two clusters: one cluster comprised the control transect (C1) while the other included some T1a and b populations (Figs. 1 and 2). The non-transect *A. d. dominicensis* site comprised individuals, or proportions of individuals, assigned to either one of the two clusters found along T1a and b. We found three clusters within *A. d. ignigularis*: one cluster included some sites along T2, one included some sites sampled along T1a and b, while the other included the *A. d. ignigularis* control transect (C3) (Figs. 1 and 2). The non-transect *A. d. ignigularis* site looked similar to sites found along T3, whereby it comprised individuals, or proportions of individuals, assigned to either cluster found along C3 and C4. All *A. d. ravitergum* sampled were initially assigned to the same genetic cluster (T2, C2 and the non-transect *A. d. ravitergum* site) (Figs. 1 and 2). Additional STRUCTURE analyses showed further substructure into two clusters: C2 comprised one cluster, while T2 and the non-transect site were assigned to another cluster (results not shown). Further STRUCTURE analyses differentiated T2 *A. d. ravitergum* from the non-transect site.

## Discussion

Our study examining numerous transitions in adaptive dewlap coloration in *Anolis distichus* across the Dominican Republic suggests that adaptive trait divergence is associated with both speciation and local adaptation in the face of gene flow. We find evidence for local adaptation at two transects (T3 and T4), whereby there was no evidence of genetic differentiation despite bimodal phenotypic variation. Patterns of mtDNA variation suggest that introgression is occurring and nuclear variation at microsatellite loci does not show evidence of any genetic structure. This is further supported by pairwise estimates of Φ_ST_ and D_PS_ that indicate similar levels of pairwise genetic differentiation to that found along the control transects. Despite the homogenizing effects of gene flow, the maintenance of dewlap color variation at such a fine spatial scale suggests that strong divergent natural selection is acting on loci underlying dewlap color differences between spatially close sites while neutral regions of the genome are homogenized with gene flow. This also suggests that the genes underlying dewlap color likely have different evolutionary trajectories that is not reflected by the results of our analyses of neutral markers. Together, these results support the importance of divergent natural selection, rather than neutral processes, in driving and maintaining dewlap color variation along these transects. These transects are similar to previous studies that have shown that adaptive trait divergence between populations can be maintained in the face of gene flow (e.g. [57–60]), including cases of adaptive color divergence [8–10, 61, 62]. It is also possible that while we did not find evidence of genetic differentiation with our markers, such ecologically-based trait divergence may represent the initial step towards speciation [63–66].

We find patterns consistent with the expected signature of speciation along three transects (T1a, T1b and T2) whereby adaptive dewlap color divergence is associated with genetic differentiation. The results of our control transects, showing no genetic differentiation where there was no change in dewlap color, further support the possibility of speciation occurring at these three transitional transects. The transects, however, exhibited differing levels of genetic differentiation and therefore likely represent different stages along the speciation continuum. T1a and b appear to be at an earlier stage along the speciation continuum relative to T2. Along T1a and b, phylogenetic and STRUCTURE analyses show distinct mtDNA clades and nuclear genetic clusters on either side of the transect, respectively. Both mtDNA and nuclear markers, however, suggest a low reduction in gene flow between the phenotypically divergent populations: mtDNA haplotypes prevalent on the western side of the transect were still found at a low frequency in the middle and other side of the transect, and nuclear markers showed evidence of admixed genotypes that decreased in frequency from the middle of the transect to the east. Furthermore, pairwise genetic distance estimates (Φ_ST_ and D_PS_) exhibit similar levels to that found along transects that show no genetic differentiation (T3, T4 and all control transects). Mitochondrial haplotype structure has been shown to reflect historic geological events while the more quickly evolving microsatellite loci are more likely to reflect contemporary gene flow [11, 67–69]. As such, the deeply divergent haplotypes we sampled from either side of the transects suggest hybridization upon recent secondary contact between *A. d. dominicensis* and *A. d. ignigularis*. While we know that these two subspecies along T1a were once isolated by a seawater channel that separated the Samaná Peninsula from mainland Hispaniola [20, 70], we are unaware of any historical barrier that isolated subspecies along T1b.

Closer to the other end of the speciation continuum, T2 exhibits evidence of a stronger reduction in nuclear gene flow; Bayesian clustering analyses suggest that hybridization is limited to one site (T2-2.5) whereby both parental genotypes and admixed individuals co-occur, and pairwise distance estimates between T2-2 and T2-2.5 are at least 3 times higher than that found along any other transect, despite closely related mtDNA haplotypes showing evidence of introgression. This pattern was also shown in Ng and Glor [20], but evidence of hybridization was only found with the additional sampling we include in this study. Our results showing high levels of genetic differentiation at nuclear loci along T2 is further supported by a previous phylogenetic reconstruction based on seven nuclear genes that showed that the same two subspecies collected at some of the same localities each formed monophyletic groups [19]. Together, this suggests that the two subspecies along T2 represent independently evolving lineages. The maintenance of a narrow hybrid zone, with no evidence of hybrids at T2-2 or T2-3 (0.89 and 1.55 km away, respectively) suggests strong intrinsic or extrinsic selection against hybrids and almost complete reproductive isolation between *A. d. ravitergum* and *A. d. ignigularis*.

While three transects support the predictions of speciation, it remains unknown whether any of the transitional transects we investigated in this study will progress to speciation, or whether the current stages we observe represent stable states of migration-selection equilibria [71]. The presence of admixed individuals at all transitional transects suggest that some level of gene flow is still occurring, and whether they progress to speciation will likely depend on the strength of divergent selection on dewlap coloration and/or whether divergent selection is also acting on other traits involved with fitness (‘multifarious selection’) (reviewed in [72]). Given that the degree to which dewlap color diverged across transects did not correlate with genetic divergence, it is likely that other traits are also involved in reproductive isolation and speciation in *A. disitchus* and that the dewlap does not serve as a ‘magic trait’ [66, 73]. For example, the abrupt reduction in gene flow across T2 was accompanied by a gradual shift in dewlap color, while T4, which showed the most abrupt shift in orange in the dewlap, did not show any genetic structure. Instead, our evidence of some genetic differentiation along T1a, b and T2, suggests that the reduction in gene flow may be more directly associated with another adaptive trait not quantified in this study. One such trait could be scale differences, which were also used to designate *A. distichus* subspecies [16]. Differences in scale number or size has been shown to vary within and among other anole species, and may represent adaptive divergences to regulate water loss or body temperature in different habitats [62, 74, 75]. If weak selection on multiple traits can promote speciation [76], this may be one reason why the polymorphic *A. d. favillarum* along T4, which only differs in dewlap color, does not show genetic structure, despite exhibiting the most abrupt dewlap color shift of all transects.

## Conclusions

Our analysis of multiple replicates of dewlap color divergence in *A. distichus* showed that geographic variation in adaptive dewlap color is associated with both local adaptation and speciation. At some transitional transects, there appears to be strong selection maintaining adaptive dewlap coloration in the face of gene flow. At other contact zones, we found signatures of speciation whereby adaptive dewlap divergence is associated with genetic differentiation, indicating some reduction in gene flow across the transects. Our results are consistent with previous work suggesting that within-island speciation in anoles is limited to the four large Greater Antillean islands [77]. Despite striking adaptive variation in head, body and dewlap coloration in anoles on the smaller islands of the Lesser Antilles, there are no examples of phenotypically divergent populations on these islands that show as strong a reduction in gene flow as we have found between *A. d. ravitergum* and *A. d. ignigularis* across T2 [9, 11, 68].

## Additional file

Additional file 1: Figure S1. Mitochondrial haplotype network comprising haplotypes from all transect sites. **Figure S2.** Pairwise estimates of D_PS_ in microsatellite loci between sites sampled along transitional transects (T1-T4; unfilled shapes) and control transects (C1-C4; filled shapes). D_PS_ estimates were divided by geographic distance (km) to correct for differing distances between sites. **Table S1.** Descriptions of the transitional transects, control transects and non-transect sites sampled for this study. For each transect, the *Anolis distichus* subspecies sampled, the length (calculated as distance from first to last site) and the number of study sites are reported. Abbreviations for *A. distichus* subspecies are as follows: *dom* = *dominicensis*, *ign* = *ignigularis*, *prop* = *properus*, *rav* = *ravitergum*, *fav* = *favillarum*. For each study site, the number of sampled individuals and observed (H_o_) and expected (H_E_) heterozygosities are described. Bold text indicates significant departures from Hardy-Weinberg equilibrium after Bonferroni correction (*P* < 0.007). **Table S2.** Estimates of mitochondrial genetic diversity for each sampled transect population. Subspecies abbreviations are as described in Table S1. (DOC 2030 kb)
