# Supplementary material for: Adaptive signal coloration maintained in the face of gene flow in a Hispaniolan Anolis Lizard
Source: BMC Evol Biol. 2016 Sep 20;16:193. doi: 10.1186/s12862-016-0763-4 (PMC5029017; doi:10.1186/s12862-016-0763-4)
Supplement: Additional file 1: Figure S1. — Mitochondrial haplotype network comprising haplotypes from all transect sites. Figure S2. Pairwise estimates of DPS in microsatellite loci between sites sampled along transitional transects (T1-T4; unfilled shapes) and control transects (C1-C4; filled shapes). DPS estimates were divided by geographic distance (km) to correct for differing distances between sites. Table S1. Descriptions of the transitional transects, control transects and non-transect sites sampled for this study. For each transect, the Anolis distichus subspecies sampled, the length (calculated as distance from first to last site) and the number of study sites are reported. Abbreviations for A. distichus subspecies are as follows: dom = dominicensis, ign = ignigularis, prop = properus, rav = ravitergum, fav = favillarum. For each study site, the number of sampled individuals and observed (Ho) and expected (HE) heterozygosities are described. Bold text indicates significant departures from Hardy-Weinberg equilibrium after Bonferroni correction (P < 0.007). Table S2. Estimates of mitochondrial genetic diversity for each sampled transect population. Subspecies abbreviations are as described in Table S1. (DOC 2030 kb) [file 12862_2016_763_MOESM1_ESM.doc]

**SUPPLEMENTARY MATERIAL**

**Figure S1.** Mitochondrial haplotype network comprising haplotypes from all transect sites.


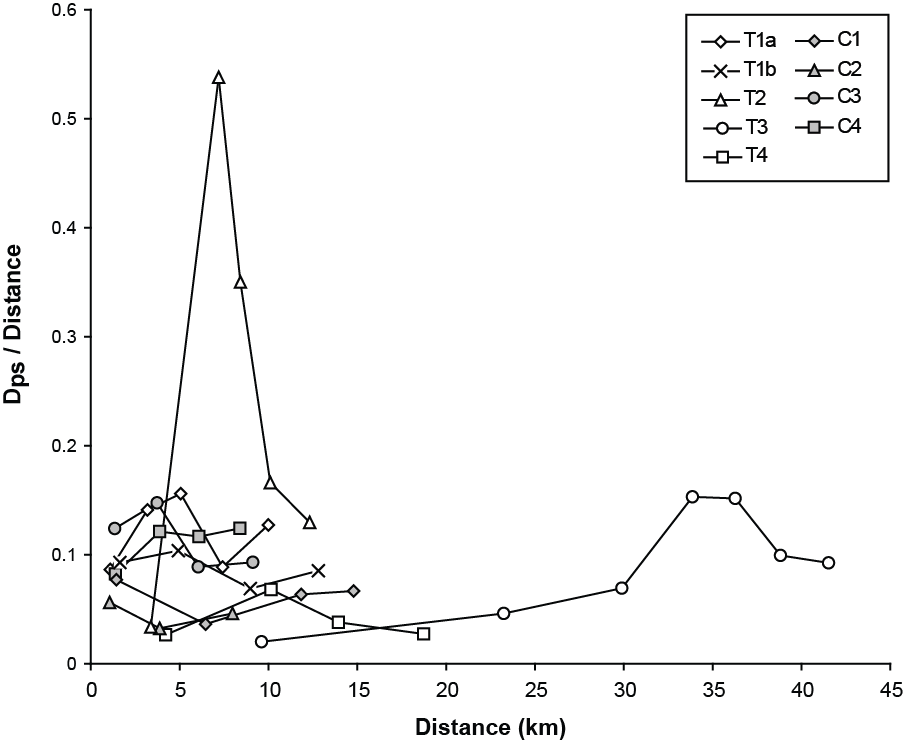


**Figure S2.** Pairwise estimates of D_PS_ in microsatellite loci between sites sampled along transitional transects (T1-T4; unfilled shapes) and control transects (C1-C4; filled shapes). D_PS_ estimates were divided by geographic distance (km) to correct for differing distances between sites.

**Table S1.** Descriptions of the transitional transects, control transects and non-transect sites sampled for this study. For each transect, the *Anolis distichus* subspecies sampled, the length (calculated as distance from first to last site) and the number of study sites are reported. Abbreviations for *A. distichus* subspecies are as follows: *dom* = *dominicensis*, *ign* = *ignigularis*, *prop* = *properus*, *rav* = *ravitergum*, *fav* = *favillarum*. For each study site, the number of sampled individuals (N), number of dewlaps quantified using a spectrometer (N dewlap), and observed (H_o_) and expected (H_E_) heterozygosities are described. Bold text indicates significant departures from Hardy-Weinberg equilibrium after Bonferroni correction (*P*<0.007).

**Supplementary Table S2.** Estimates of mitochondrial genetic diversity for each sampled transect population. Subspecies abbreviations are as described in Table S1.
